# High H_2_O_2_ Utilization Promotes Selective Oxidation of Methane to Methanol at Low Temperature

**DOI:** 10.3389/fchem.2020.00252

**Published:** 2020-04-07

**Authors:** Yang Yan, Changlei Chen, Shihui Zou, Juanjuan Liu, Liping Xiao, Jie Fan

**Affiliations:** ^1^Department of Chemistry, Zhejiang University, Hangzhou, China; ^2^College of Materials & Environmental Engineering, Hangzhou Dianzi University, Hangzhou, China

**Keywords:** methane oxidation, AuPd colloid, H_2_O_2_ utilization, pH, Au-to-Pd ratio, low temperature

## Abstract

Selective oxidation of methane to methanol has been often considered as a “holy grail” reaction in catalysis. Herein, we systematically investigate the effect of solution pH and Pd-to-Au ratio of AuPd_*x*_ colloid on the catalytic performance of methane oxidation. It is revealed that these two parameters can determine the amount of H_2_O_2_ participated in the reaction, which is linearly related to the productivity of oxygenates. A high catalytic performance in methane activation requires a high utilization of H_2_O_2_ to generate more ·OH. The optimal pH is 3.0 and the optimal Pd-to-Au ratio is between 0.1 to 0.7.

## Introduction

Natural gas, the cleanest and cheapest fossil fuel, is widely accepted as a promising alternative resource to crude oil. According to BP statistical review of world energy, the proven natural gas reserve is more than 190 trillion cubic meters in 2018. Its global consumption and production have increased by over 5%, which recorded the largest annual growth for over 30 years (Dudley, 2019). Unfortunately, natural gas reserve is generally located in remote areas far away from its markets, which leads to high transportation cost (Jones et al., 1987). In order to economically utilize the resource, it is necessary to convert methane, the major constituent of natural gas, into liquid fuels for easier transportation. Currently, the industrial transformation of methane proceeds via an indirect route in which methane is first converted to syngas and then the syngas is converted to methanol and higher hydrocarbons (Vernon et al., 1990; Cheng et al., 2016, 2017; Jiao et al., 2016; Alvarez-Galvan et al., 2019). The high energy consumption and capital input, however, limit the wide application of this process, especially in the utilization of remote located natural gas fields. To address this problem, increasing effort has been devoted to the direct conversion of methane, a more economical and environmentally friendly route to utilize methane (Tang et al., 2014; Schwach et al., 2017). Among them, the direct oxidation of methane to methanol has been one of the major challenges for many decades (Palkovits et al., 2009; Grundner et al., 2015; Shan et al., 2017; Sushkevich et al., 2017; Cui et al., 2018; Park et al., 2019; Jin et al., 2020).

In practice, the oxidation of methane to methanol can be performed in the liquid or gas phase (Palkovits et al., 2009; Sushkevich et al., 2017; Cui et al., 2018; Park et al., 2019). However, the gas-phase and high temperature processes generally have limited methanol yield due to the thermodynamically favored over-oxidation of methanol to CO_x_. During the last few years, increasing attention has been devoted to the low-temperature liquid-phase oxidation of methane to methanol using N_2_O, O_2_, and H_2_O_2_ as oxidants (Agarwal et al., 2017; Williams et al., 2018; McVicker et al., 2020). Recently, Hutchings et al. reported a low-temperature (50°C) route in aqueous hydrogen peroxide (H_2_O_2_) for oxidizing methane to methanol in high yield (92%). By using unsupported colloidal Au-Pd nanoparticles as a catalyst, they successfully incorporated molecular oxygen into the liquid oxidation products (Agarwal et al., 2017). It is important to highlight that the activation of methane to methyl radical by H_2_O_2_ is a key step for the incorporation of molecular oxygen. However, no quantitative relationship between the utilization of H_2_O_2_ and methane activation activity is obtained yet. Besides, due to the self-decomposition of H_2_O_2_ in the reaction conditions, only part of H_2_O_2_ can participate in the activation of methane. Considering the high price of hydrogen peroxide (0.67 $ kg^−1^ of 100% H_2_O_2_) (Thomas et al., 2000), it is also economically important to maximize the utilization efficiency of H_2_O_2_. To address these problems, we tightly control the solution pH and Pd-to-Au ratio of AuPd colloid nanoparticles to manage the utilization efficiency of H_2_O_2_. It turns out that the productivity of oxygenates is linearly related to the amount of H_2_O_2_ that participated in the activation of methane.

## Experimental

### Chemicals and Materials

Sodium borohydride (NaBH_4_, 96%), palladium chloride (PdCl_2_, 98.5%), hydrogen tetrachloroaurate trihydrate (HAuCl_4_·3H_2_O, 99.9%) were obtained from Sinopharm Chemical Reagent Co., Ltd., China. Polyvinyl pyrrolidone (PVP, 360,000 Da) was from Shanghai Macklin Biochemical Co., Ltd., China. Methane (CH_4_, 99.99%) and oxygen (O_2_, 99.999%) was from Hangzhou Jingong Gas, China. Deionized water was used throughout the experiments.

### Catalyst Preparation

The colloidal AuPd_*x*_ nanoparticles (*x* is the Pd-to-Au molar ratio) were synthesized by a deposition (Lopez-Sanchez et al., 2008; Agarwal et al., 2017) method. Firstly, Au and Pd precursor were prepared in deionized water with polyvinyl pyrrolidone (PVP, 360,000 Da) added as a stabilizer. After 10 min of stirring, the precursors were reduced with freshly prepared 0.1 M NaBH_4_ solution. The red or brown colloid was left stirring for at least 30 min to ensure all the metal precursor salts reduced to metallic nanoparticles and NaBH_4_ decomposed.

### Characterizations

Transmission electron microscopy (TEM) images were taken using a Hitachi HT7700 microscope operated at 120 kV by drop casting the nanocrystal dispersions onto carbon-coated Cu grids and drying under ambient conditions. Powder X-ray diffraction (XRD) patterns were recorded on a Rigaku Ultima IV diffractometer with Cu Kα radiation. UV-vis absorption spectra were taken using a UNIC UV-2802 spectrophotometer.

### Catalytic Measurement

The oxidation of methane was conducted using a stirred autoclave reactor (30 mL, DHA-M630). Typically, the vessel was charged with 10 mL of catalyst (Au-Pd colloid, 6.6 μmol) and fresh added H_2_O_2_. The pH of the solution was adjusted by HCl and NaOH. Subsequently, the autoclave was sealed and purged 3 times with methane before being pressurized with methane (3.0 MPa) and oxygen (0.5 MPa). The autoclave was heated to 50°C within 20 min and stirred at 700 rpm for another 30 min. Once the reaction was completed, the autoclave was put into an ice bath. The concentration of H_2_O_2_ was quantified by UV-vis spectroscopy with acidified K_2_TiO(C_2_O_4_)_2_ solution as chromogenic agent (Meng et al., 2018; Wang et al., 2019). The gas phase product of the reactions was quantified by GC with TCD detectors. ^1^H-NMR studies were carried out to quantify the amounts of liquid phase products using a Bruker 600 MHz NMR equipped with a solvent suppression system (Shan et al., 2017). An internal standard containing 1% TMS in CDCl_3_ (99.9% D) was placed in a sealed tube and used to quantify the amount of product. It is important to highlight that the presence of PVP is very important to the stability of AuPd colloid catalysts. TEM images of spent catalysts under different pH suggest that the particle sizes of the spent catalysts are close to each other and are only slightly larger than those of fresh catalysts, indicating the colloidal catalysts are stable in the pH range from 1 to 8. In contrast, colloidal catalysts without PVP coagulate after 3 h reaction at pH 6–8 or 0.5 h reaction at pH 1–5.

## Results

The methane oxidation reaction was carried out in a stirred autoclave reactor using O_2_ and H_2_O_2_ as oxidants to gain methyl hydroperoxide and methanol as primary products in aqueous solution at 50°C. Other products included formic acid and CO_x_ was scarcely detected. AuPd_*x*_ nanoparticles (*x* is the Pd-to-Au molar ratio) synthesized by a typical colloidal method were utilized as catalysts (Lopez-Sanchez et al., 2008; Agarwal et al., 2017). Two key parameters, the solution pH and the Pd-to-Au ratio, were investigated to reveal the quantitative relationship between H_2_O_2_ utilization and the activity of methane activation. The concentration of H_2_O_2_ after reaction was determined by a spectrophotometry method with Ti reagent (Wang et al., 2019). It is important to note that due to the self-decomposition of H_2_O_2_, only part of the H_2_O_2_ are involved in the activation of methane. The self-decomposition of H_2_O_2_ was determined by control experiments that conducted under the same reaction conditions without methane. The amount of H_2_O_2_ participated in methane activation (denoted as reactive H_2_O_2_) is calculated by the difference between the amount of total consumed H_2_O_2_ and the self-decomposed H_2_O_2_.

### The Effect of pH on CH_4_ Oxidation

**Figure 1:**
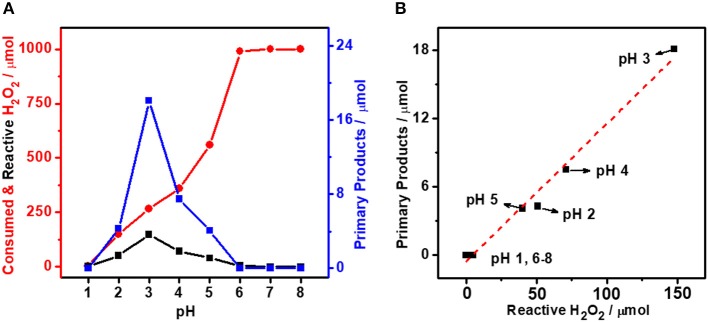
**(A)** Methane oxidation reaction performance carried out with AuPd_0.1_ colloid with various pH. **(B)** Primary products as a function of reactive H_2_O_2_ with various pH. Reaction conditions: 3.0 MPa methane, 0.5 MPa O_2_, 1,000 μmol H_2_O_2_, 50°C, 0.5 h, 6.6 μmol metal per reaction.

Solution pH is a key parameter that affects the lifetime of H_2_O_2_ (Samanta, 2008; Jung et al., 2009). In this work, the initial pH of reaction solution was adjusted from 1.0 to 8.0. Table 1 shows the H_2_O_2_ consumption under different reaction conditions. Notably, the H_2_O_2_ decomposition in the absence of AuPd colloid is very similar to that with AuPd colloid, indicating the self-decomposition of H_2_O_2_ (unselective H_2_O_2_ decomposition) is mainly determined by the reaction condition (solution pH) though the presence of AuPd colloid also increases the self-decomposition of H_2_O_2_. Figure 1A plots the amount of total consumed H_2_O_2_ and reactive H_2_O_2_ as functions of solution pH. Interestingly, these two plots are very different from each other. The total consumption of H_2_O_2_ increases rapidly as the pH. At pH = 1, only trace of H_2_O_2_ is consumed while at pH higher than 6, all H_2_O_2_ is consumed. On the contrary, the reactive H_2_O_2_ exhibits a volcano plot with pH. The maximum amount of reactive H_2_O_2_ is achieved at pH = 3. These results suggest that a proper pH is crucial to transform H_2_O_2_ for methane activation. At low pH, the H_2_O_2_ is too stable to active methane. At high pH, the H_2_O_2_ decompose too fast so that its participation in methane activation is also limited.

**Table 1:** H_2_O_2_ consumption with/without AuPd_0.1_ colloid.

**Reaction condition**	**H**_****2****_**O**_****2****_ **consumption/μmol**							
	**pH = 1**	**2**	**3**	**4**	**5**	**6**	**7**	**8**
Without AuPd_0.1_	0	36	64	192	350	860	920	1,000
With AuPd_0.1_	0	99	118	289	520	985	1,000	1,000
With AuPd0.1 and CH_4_	0	150	266	360	560	990	1,000	1,000
Reactive H_2_O_2_	0	51	148	71	40	5	0	0

Furthermore, we also plot the amount of oxygenates as a function of pH. Notably, it shows a similar volcano trend with the reactive H_2_O_2_. The maximum productivity (22.9 μmol of total products and 18.1 μmol of primary products) is also achieved at pH = 3. To quantitatively analyze the relationship between methane activation activity and the utilization of H_2_O_2_, we further plot the amount of oxygenates as a function of the amount of reactive H_2_O_2_. As shown in Figure 1B, a linear relationship is obtained, which clearly reveal the decisive role of reactive H_2_O_2_ in the activation of methane. It is indicated that a proper acidity of reaction environment is crucial to maximize the utilization of H_2_O_2_ for more efficient oxidation of methane to methanol.

### The Effect of Au-Pd Ratio on CH_4_ Oxidation

**Figure 2:**
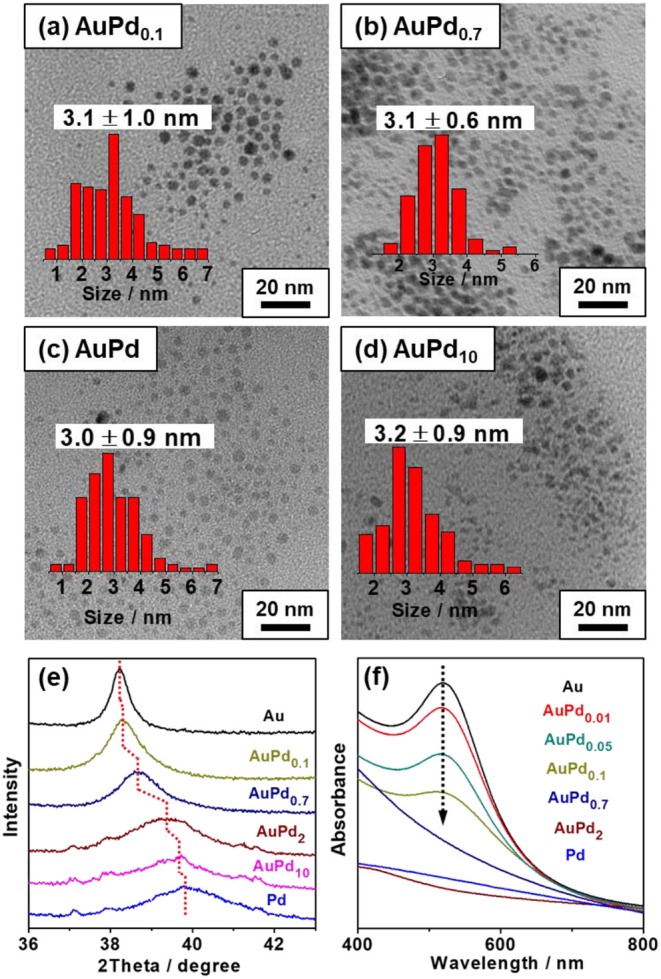
TEM images and particle size distributions of colloidal AuPd_*x*_ nanoparticles. **(a)** AuPd_0.1_, **(b)** AuPd_0.7_, **(c)** AuPd_1.0_, **(d)** AuPd_10_, **(e)** XRD, and **(f)** UV-vis spectrum of colloidal AuPd_x_ catalysts.

It is generally accepted that Au is less active than Pd toward H_2_O_2_ decomposition (Choudhary et al., 2006; Li et al., 2011). Our previous study (Yan et al., 2020) suggests that the Au can alter the electronic structure of Pd in bimetallic catalysts to influence their catalytic performance. The tuning of Pd-to-Au ratio in AuPd_x_ is therefore expected to regulate the self-decomposition and thus the utilization efficiency of H_2_O_2_. Figures 2a–d show the TEM images of colloidal AuPd_*x*_ nanoparticles. As can be seen from these figures, AuPd_*x*_ with different Pd-to-Au molar ratios share the similar spherical morphology and particle size (~3 nm). According to the literatures (Pritchard et al., 2013; Agarwal et al., 2017), AuPd nanoparticles synthesized by colloidal method are bimetallic alloys. The surface electronic structure of AuPd colloid was investigated by XPS in these literatures (Agarwal et al., 2017; McVicker et al., 2020). It turns out that Au and Pd are mainly in their metallic states. Electrons are transferred from Pd to Au, consistent with their electronegativities (Au, 2.54; Pd, 2.20). The charge interaction increases the Au s-state occupancy and indicates the bimetallic alloy formation (Chen et al., 2018; Yuan et al., 2018). In order to verify the formation of AuPd alloy, we herein collect XRD patterns for colloidal AuPd_*x*_ nanoparticles. As shown in Figure 2e, all AuPd_*x*_ nanoparticles exhibit symmetrical peak between Au (111) and Pd (111). Besides, as the Pd-to-Au ratio increases, the diffraction peak of AuPd_*x*_ nanoparticles shifts continuously to a higher angle toward the Pd (111) peak. These results clearly confirm that all AuPd_*x*_ nanoparticles are single-phase AuPd alloy (Qiao et al., 2014). In addition, the characteristic Au plasmon resonance band (~ 520 nm) decreases with the increasing of palladium content and finally disappears, suggesting the changes in band structure and the alloying of Au and Pd (Figure 2f) (Deki et al., 1999).

**Figure 3:**
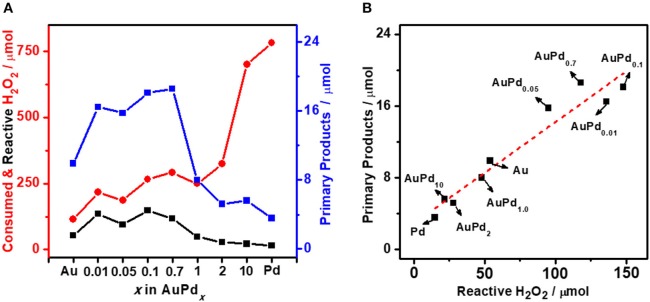
**(A)** Methane oxidation reaction performance carried out with colloidal AuPd_*x*_ nanoparticles. **(B)** Primary products as a function of reactive H_2_O_2_ with various *x* (Au-to-Pd ratio). Reaction conditions: 3.0 MPa methane, 0.5 MPa O_2_, 1,000 μmol H_2_O_2_, 50°C, 0.5 h, initial pH = 3.0, 6.6 μmol metal per reaction.

Figure 3A plots the amount of total consumed H_2_O_2_ and reactive H_2_O_2_ as functions of Pd-to-Au ratio. A distinct increase in H_2_O_2_ total consumption is observed from 115 to 781 μmol with the increasing of palladium content (*x* value of AuPd_*x*_) of the colloid catalyst, confirming Pd is more active than Au for H_2_O_2_ decomposition. In terms of reactive H_2_O_2_, a volcano curve is observed. The platform of high reactive H_2_O_2_ amount is achieved when the *x* of AuPd_*x*_ is between 0.1 and 0.7. Further increasing Pd-to-Au ratio would significantly increase the self-decomposition of H_2_O_2_ and therefore decrease the amount of reactive H_2_O_2_. It is important to note that the amount of primary oxygenates produced on AuPd_x_ also follows the similar trend as reactive H_2_O_2_, with AuPd_0.1_ to AuPd_0.7_ showing much higher activity than other catalysts. To quantitatively analyze the relationship between methane activation activity and the utilization of H_2_O_2_, we further plot the amount of primary oxygenates as a function of the amount of reactive H_2_O_2_ over various AuPd_*x*_ catalysts. The quasi-linear relationship between reactive H_2_O_2_ and primary products (Figure 3B) again reveals the decisive role of reactive H_2_O_2_ in the activation of methane. It is interesting to note that the slope in Figure 3B (0.11) is almost identical to that in Figure 1B (0.12), suggesting that both pH and the Au-Pd ratio affect the H_2_O_2_ utilization and methane oxidation in a similar way. A proper Au-Pd ratio in catalyst is crucial to transform H_2_O_2_ for methane activation. Specifically, Au is less active to transform H_2_O_2_ while Pd is too active for the decomposition of H_2_O_2_. Medium Pd-to-Au ratios, i.e., 0.1–0.7, can successfully balance the activation and self-decomposition of H_2_O_2_. They transform most H_2_O_2_ to active methane, which leads to maximum productivity of oxygenates.

## Discussion

Previous study by Hutchings et al. (Agarwal et al., 2017) suggested that the activation of CH_4_ by H_2_O_2_ is the rate-determining step for the mild oxidation of methane of methanol. It proceeds through a radical mechanism with ·OH radicals generated from H_2_O_2_ serving as the radical initial agent to activate CH_4_ to CH_3_·. Once CH_3_· radicals were formed, they readily react with O_2_ to form CH_3_OH. It is important to note that the generation of ·OH from H_2_O_2_ is dependent on the solution pH (Maezono et al., 2011). At acidic solution (pH <4), H_2_O_2_ is relative stable and trends to generate ·OH whereas at basic solution, H_2_O_2_ decomposes very fast to generate O_2_ and H_2_O (Barreiro et al., 2007). In this study, the optimal pH for methane activation is 3.0, the same as that for the generation of ·OH (Maezono et al., 2011). Taken together, the linear relationship between reactive H_2_O_2_ and oxygenates productivity could be linked by the generation of ·OH. The more easily ·OH radicals are generated, the more amount of oxygenates are produced. In terms of Pd-to-Au ratios, the addition of Au into Pd can dilute the surface to reduce the number of sites for O-O scission. Besides, the electron transfer between Au and Pd also alter the adsorption strength of H_2_O_2_ with metal surface (Kanungo et al., 2019). A relative mild decomposition of H_2_O_2_ to ·OH radicals can therefore be achieved by tuning the Pd-to-Au ratio. Similar results have been reported in the direct synthesis of H_2_O_2_ from H_2_ and O_2_ (Kanungo et al., 2019). According to the literature, a high concentration of ·OH radicals facilitates the disproportionation to H_2_O and O_2_ via a hydrogen transfer mechanism (Plauck et al., 2016). To this end, the regulation of Pd-to-Au ratio can affect the generation and transformation of ·OH, which in return quasi-linearly affects the productivity of oxygenates. It is important to note that at the reaction condition we utilized in this study, the gain factor, defined as mol of oxygenate produced/mol of reactive H_2_O_2_, is <1. This result indicates that only part of ·OH radicals participates in the activation of methane. The rest of them likely disproportionate to form H_2_O and O_2_ via a hydrogen transfer mechanism (Plauck et al., 2016). Hutchings et al. suggested that reducing the addition of H_2_O_2_ could significantly increase the gain factor to 1.2 (Agarwal et al., 2017). It is most likely because decreasing the addition of H_2_O_2_ can significantly decrease the amount and therefore the disproportionation of ·OH. To this end, an ideal way to utilize H_2_O_2_ for methane activation is to *in-situ* generate H_2_O_2_. Interestingly, this idea is recently realized by Jin et al. (2020). By utilizing a hydrophobically coated zeolite as the catalyst, they successfully generated peroxide from H_2_ and O_2_ and keep it close to the AuPd active site, where incoming methane is selectively oxidized to methanol.

## Conclusion

We systematically investigated the influence of solution pH and Pd-to-Au ratio of AuPd colloid nanoparticles on the catalytic performance in methane oxidation to methanol. Linear relationships were obtained between the reactive H_2_O_2_ and the productivity of oxygenates, demonstrating improved H_2_O_2_ utilization efficiency is critical for methane activation. Developing catalysts that can *in situ* generate H_2_O_2_ (or ·OH) and active methane is a promising direction to produce methanol from methane.

## Data Availability Statement

All datasets generated for this study are included in the article/supplementary material.

## Author Contributions

JF and SZ designed the study. YY and CC performed most of the experiments. YY, CC, SZ, and JF wrote the paper. JL and LX performed some of the experiments and revised the paper.

### Conflict of Interest

The authors declare that the research was conducted in the absence of any commercial or financial relationships that could be construed as a potential conflict of interest.
